# Factors Affecting the Job Satisfaction of Caregivers in a Home-Based Elderly Care Program

**DOI:** 10.3390/ijerph19159332

**Published:** 2022-07-30

**Authors:** Xiao Rong, Zhipeng Zhou, Yihui Su

**Affiliations:** 1 Department of Architecture, Shanghai University, No. 149 Yanchang Road, Shanghai 200072, China; xiaorong17@shu.edu.cn; 2 Department of Sociology, Cornell University, Ithaca, NY 14850, USA; zz334@cornell.edu; 3 Department of Sociology, College of Humanities, Shanghai University of Finance and Economics, No. 777 Guoding Road, Shanghai 200433, China

**Keywords:** home-based elderly care, in-home elderly caregiver, job satisfaction, social insurance

## Abstract

Population aging has increased the demand for elderly care worldwide. The home-based elderly care system plays an important role in meeting this demand in developing countries. The quality of home-based elderly care is associated with the job satisfaction of caregivers in home-based elderly care programs, which has rarely been studied. This paper explores the factors that affect the job satisfaction of these elderly caregivers, including personal characteristics, working conditions, employment status, training, caregiver–client relationships, welfare, work experience, and burnout. It utilizes data from the Shanghai Domestic-work Professionalization Survey (SDPS), which was conducted among four types of in-home caregivers (*n* = 1000) in Shanghai over the period from May to September 2021. This paper selected a sample of elderly caregivers (*n* = 285) to examine their job satisfaction. The results show that gender, age, marital status, how they earned the job, relation with clients, social insurance, and work experience are significantly associated with the job satisfaction of in-home elderly caregivers, and their job satisfaction is negatively associated with their burnout levels. However, training and working conditions have no significant effect on the job satisfaction of in-home elderly caregivers, which is different from previous studies on formal care workers, such as nurses, in the institutional care system.

## 1. Introduction

Population aging has become a global trend, which increases the demand for formal and informal care services for elderly people worldwide. To meet this demand, many developed countries in Europe and North America have promoted home-based elderly care, as this mode is preferred by clients [1,2]. Likewise, developing countries have also begun to provide community-based and home-based elderly care, since the traditional informal care provided by family members cannot meet the surging demand [3]. The global growth of the home-based elderly care industry has led to an increase in the number of in-home elderly caregivers in both developed and developing countries. Studies show that most in-home elderly caregivers in these countries are migrant women [4]. In contrast to formal care workers, i.e., nurses, who are well-studied as formal care workers in existing research, these in-home caregivers in the informal sector have not received enough attention from scholars [5,6,7,8,9,10]. To contribute to the current studies of care work, this paper explored the job satisfaction of elderly caregivers in home-based elderly care in developing countries, where the majority of people rely on the home-based elderly care system and the care work of in-home elderly caregivers.

China is a typical rapidly aging developing country. According to the United Nations, the number of people aged 65 years or over in China accounted for 11.5% of the total population in 2019, and this proportion is expected to reach 16.9% by 2030 [11]. A variety of services is required to meet the needs of the increasing elderly population. In particular, China relies on the home-based care system to meet the rapid growth in demand for elderly care [12]. Although the public institutional care system is gaining prominence, home-based care is still the dominant form of elderly care in China [13]. Due to the influence of Confucian tradition, Chinese seniors prefer to spend their remaining years at home rather than in nursing homes [14,15]. A study also shows that only 1.16% of Chinese elderly people are covered by institutional care, while 98.84% still depend on in-home care [16]. Even long-term care in China is mainly home- and community-based and is provided through commercial and noncommercial community-based agencies [17]. Studies show that at least 10 million elderly care workers in the long-term care system have been trained and sent to urban families to provide service [18,19]. Therefore, in-home caregivers play a significant role in the elderly care system in China. These caregivers are formal and informal care workers employed by domestic service companies or agencies financed by the local government.

With regard to home-based elderly care, current research revolves around whether the need has been met but has concentrated less on the quality of care [20,21,22,23,24]. However, the quality of care provided by the worker is key to guaranteeing older people’s wellbeing and the quality of their daily life. Moreover, scholars find that the quality of elderly care is closely related to caregivers’ job satisfaction [25,26]. Caregivers’ job satisfaction refers to the extent to which a caregiver is satisfied with his or her job, reflecting their personal perception towards work and work experiences [27]. Previous studies have shown that caregivers with higher job satisfaction tend to have better job performance, which also have a positive effect on work commitment and workers’ intent to remain in their current professions [28]. Caregivers’ higher job satisfaction has been associated with higher efficiency and better quality of care service as well as higher care receiver satisfaction [29,30]. Furthermore, researchers find that job satisfaction is also directly and indirectly associated with their feelings of burnout, which refers to a psychological phenomenon including emotional exhaustion, depersonalization, and feeling of lacking in personal accomplishment [31]. To measure the burnout from these three dimensions, Maslach and Jackson developed the Maslach Burnout Inventory (MBI), which is frequently used in research on caregivers’ burnout and job satisfaction [31]. With the MBA toolkit, researchers found that a complex relationship exists between caregivers’ burnout and job satisfaction. Some scholars assert that burnout is a predictor of job satisfaction [10,32], while others argue that low job satisfaction might result in occupational burnout [33,34,35]. Both sides agree that high job satisfaction is associated with low burnout level [36,37]. 

Existing studies have examined the influence of multiple factors on caregiver job satisfaction, including personal characteristics, working conditions, training, and caregiver–client relationships. These studies have found that personal characteristics, such as gender, age, race, marital status, and education, have less of an effect on job satisfaction [38,39,40,41]. In contrast, working conditions play a more important role [42], among which the payment and benefit they receive, the length of working hours, work demands, promotion, supervision they face, and social relations with their colleagues all affect their job satisfaction [43,44,45,46,47]. It has been found that better working circumstances, such as opportunities for professional development and participation in decision-making, empower caregivers, contributing to their perception of support [48], which is beneficial for job satisfaction, resulting in positive job performance and high-quality care for care receivers [18]. Furthermore, studies show that training programs might improve caregivers’ job satisfaction [49,50]. The information and technology received in training programs might improve caregivers’ perception of support for their work. Moreover, the social relationship and interaction between caregivers and elderly people also affect caregivers’ satisfaction and attitude [51,52]. Research findings show that caregivers were satisfied with their jobs if they received good feedback from the care recipients [53,54].

Although these studies have revealed the influence of personal characteristics, working conditions, training, and relationships with clients on job satisfaction, they are focused on institution-based caregivers, such as care workers in nursing homes. These findings may not be applicable for in-home elderly caregivers, because they are quite different from institution-based care workers. First, in-home elderly caregivers are mostly migrant women whose education is lower than that of institutional care workers such as nurses in the nursing home [55]. Second, the training received by in-home caregivers is less professional than that received by formal care workers. Third, their working conditions are different from those of formal care workers because their workplace, working time, and ways of promotion are unique. Furthermore, due to the COVID-19 pandemic lockdown, in-home elderly caregivers’ job opportunities have become fewer compared with institutional care workers. During the pandemic, clients tend to reject the in-home elderly caregivers’ attempts to enter their home, which interrupts these in-home caregivers’ working arrangements, making their job more precarious [56]. However, the differences between in-home elderly caregivers and institutional care workers have not been adequately explored, as there are very few empirical studies examining the factors that are associated with job satisfaction among in-home elderly caregivers. Moreover, in contrast to care workers in the institutional care system, these in-home elderly caregivers are migrants, lacking social protection and welfare, which is also the most important difference between caregivers in institutional care and those in home-based care systems. Current studies of immigrants imply that the social protection and welfare they receive plays an important role in their work and daily life [57]. However, the relationship between welfare the caregiver receives and its influence on job satisfaction has not received enough attention from scholars.

Therefore, this study explores the factors that affect the job satisfaction of in-home elderly caregivers using Respondent-Driven-Sampling of elderly caregivers (*n* = 285) from the Shanghai Domestic-work Professionalization Survey. It aims to facilitate policy makers such as Ministry of Human Recourse and Social Security of the People’s Republic of China and the local government to develop policies to promote the elderly care system in China and to protect the elderly caregivers’ social rights. It also contributes to the previous literature in two aspects. First, we focus on the job satisfaction of care workers who provide care to older people’s families rather than to nursing homes. As in-home care workers in developing countries have been under-researched, our study may contribute to the discussion on the job satisfaction of care workers in countries where home-based elderly care is the foundation. Second, our work analyzes the influence of factors such as training, working conditions, and worker–client relationships in addition to discussing the effect of social protection and welfare on care workers’ job satisfaction. In this way, our work may provide recommendations to enhance job satisfaction related to social welfare.

## 2. Materials and Methods

### 2.1. Data and Design

This study utilizes data from the Shanghai Domestic-work Professionalization Survey (CDPS). CDPS was funded by The National Social Science Fund of China and conducted from early May to early September in 2021 in Shanghai based on the Respondent-Driven-Sampling approach. The aim of this research is to identify factors which may help improve the job satisfaction of elderly caregivers in a home-based elderly care program. This could help policy makers such as Ministry of Human Recourse and Social Security of the People’s Republic of China and the local government to formulate policies to promote the quality of elderly care and guarantee the elderly caregivers’ social rights.

Three predefined respondent inclusion criteria include: (1) respondents must provide domestic work services for a private family, instead of purely for other organizations such as, but not limited to, a corporation and a government department; (2) prior to interview, respondents must have undertaken domestic work for more than three months; and (3) the geographical range of domestic work service is restricted to Shanghai. All interviews were conducted face-to-face.

Twelve initial seeds were selected based on previous fieldwork among four occupations: maternal-child caregivers (6), houseworker (1), elderly caregivers (2), and cleaners (3). Each respondent was rewarded with 50 RMB (about $7.70) for answering the questionnaire, and with 10 RMB (about $1.50) for each successful recruitment. In order to record referral chain information, each participant was assigned three coupons labeled with a specific number. In this way, CDPS acquired a total sample size of 1000, and a design effect of each characteristic greater than two, which enhances confidence in the sample. For the purpose of this study, we selected the samples who were elderly caregivers, which resulted in a size of 285.

The questionnaire was divided into seven blocks across 79 items, containing questions about personal characteristics, working conditions, employment status, job satisfaction, psychometric characteristics, use of internet, and home-based clients.

### 2.2. Variables

The personal characteristics variables included gender, age, level of education (no education completed, primary school, junior high school, high school, vocational high school, technical secondary school, college, undergraduate, and above), and marital status (married, divorced, widowed, single). In terms of working conditions, respondents were asked about annual income, number of rest days per month, and the type of care work (lived-in, day shift, hourly). With regard to employment status, respondents were inquired about the type of contract (no contract, labor contract, tripartite agreement, private agreement with clients) and how they received their job (relatives and friends, agencies, internet, others). In terms of training, respondents were asked about whether the company provided training, whether they have received training, and the number of certificates they have obtained. Respondents were also inquired about their relation with clients (very good, good, common, bad, very bad). In terms of welfare, respondents were asked about social insurances they have obtained. Lastly, in terms of work experience, respondents were acquired about in which year they started to work as in-home elderly caregivers. Some of the categories of these variables were merged in the regression model, to achieve a more balanced distribution of the sample size by category and a better model result. The merged categories are reported in the next section.

Job satisfaction included four items, scored from 1 (not at all satisfied) to 5 (very satisfied): work income, relationships with clients, professional status, and working conditions. Considering the relevance of individuals’ psychometric characteristics to job satisfaction, we examine the burnout levels of in-home elderly caregivers as a supplement to our analysis. We selected two types of items to reflect burnout. Type 1 included five items, scored from 1 (never) to 5 (always), regarding the frequency of feelings of lack of breaks, disruption of sleep, need for emotional adjustment, breakdown, and loss of interest. Type 2 included two items, scored from 1 (strongly disagree) to 5 (strongly agree), regarding whether the respondent likes the job, and whether the job can help the respondent meet more people. These items are an approximation to Maslach Burnout Inventory (MBI), although CDPS has not strictly adopted MBI.

### 2.3. Statistical Analysis

We first conducted a factor analysis on the job satisfaction variables and on the burnout variables to determine the specific factors associated with the level of job satisfaction and the level of burnout among elderly caregivers. Multiple regression models were then constructed to identify the personal and professional characteristics associated with job satisfaction followed by the correlation analysis between job satisfaction and burnout levels, as well as a supplementary multivariable analysis for burnout levels of in-home elderly caregivers. All the statistical analyses were conducted using Stata 16.

## 3. Results

Table 1 shows the descriptive analysis of the personal characteristics and employment-related status of the respondents. The majority (94%) of the respondents were women. The median age was 51 years. Almost 85% of the respondents had only completed junior high school and below. The most common marital status was married, accounting for more than 90%.

On average, the sample received ¥62,004 (equivalent to $9300) per year and rested 1.7 days per month. More than 61% of the respondents were hourly workers, and around 24% were lived-in workers. The most common ways for them to obtain their jobs were through relatives and friends (49.1%) and agencies (39.3%). Almost 55% of the respondents were in a labor contract, but almost 29% had no contract at all. Over 81% of the respondents had training experience, and the respondents had acquired 1.6 certificates on average. Nearly 59% of the respondents were working for companies which had provided training opportunities.

Regarding the relation with clients, more than 54% of elderly caregivers had a very good relationship with clients. In terms of social insurance, a total of 88% of respondents were only covered by the new rural cooperative medical insurance, and only 12% were able to enjoy the three or five insurances provided by the state and companies. The average work experience of the elderly caregivers was 6.2 years.

The results for the levels of job satisfaction are reported in Table 2. We first performed correlation analysis using the KMO and Bartlett Sphericity tests. The KMO value was 0.715 and the *p* value was below 0.05, indicating that the original variables were suitable for factor analysis and the principal component analysis (PCA) could be conducted. Furthermore, the Cronbach’s alpha was 0.6898, which provided evidence for the scale reliability. Using the varimax rotation approach with the threshold of eigenvalues of principal components as 1, we obtained one factor that captures the majority of variation in the job satisfaction of elderly caregivers in the home-based elderly care program (Table 3). The results of PCA show that the cumulative contribution ratio of the first principal component variance is 52.65%, indicating that we could use only one factor to capture most variations in individuals’ job satisfaction levels.

The psychometric characteristics related to burnout are reported in Table 4. The KMO and Bartlett Sphericity tests showed that the KMO value was 0.724, the *p* value was below 0.05, and the Cronbach’s alpha was 0.7206. Then, using the varimax rotation approach with the threshold of eigenvalues of principal component as 1, we obtained two factors that capture the majority of variation in burnout of caregivers in the home-based elderly care program (Table 5). Factor 1 is related to emotional exhaustion, and factor 2 is related to lack of personal accomplishment. The cumulative contribution ratio of the first two principal component variances was 55.61%, which indicates those two factors explain the majority variations in burnout levels.

In order to analyze the impact of each independent variable more intuitively and to enhance the interpretability, we standardized the factor scores to construct indices of job satisfaction and burnout (emotional exhaustion and lack of personal accomplishment) ranging from 0 to 100, on which the following regression analysis will be based (Table 6). The conversion adopts the following formula: Standardized factor value = (factor value − factor (min)) × 100 ÷ (factor(max) − factor(min)). 

Table 7 shows the results of two multivariable linear regression models using the index of job satisfaction dependent variable with a confidence level of 95%. Model 1 includes independent variables regarding personal characteristics, working conditions, employment status, training, relation with clients, and social insurance, while model 2 includes all these independent variables as well as work experience.

We first examined the results of model 1. In terms of personal characteristics, gender and marital status are significantly associated with job satisfaction. Men were less likely than women to have a higher level of job satisfaction, and divorced elderly caregivers were less likely than those married to have a higher level of job satisfaction. Age and the education level showed no significant association. All the independent variables regarding working conditions (annual income, number of rest days per week, the type of care work), and training (whether the company provided training, whether they have received training and whether they have obtained certificates) were not significantly associated with the level of job satisfaction. In terms of employment status, ways to find jobs were significantly associated with job satisfaction, while the type of contract showed no significant association.

However, independent variables regarding the relation with clients and the social insurance were found to be significantly associated with the level of job satisfaction. Elderly caregivers who have a very good relation with clients were more likely to enjoy a high level of job satisfaction than those who do not have a very good relation with clients. In terms of the social insurance, those who were covered by five insurances were more probable to have a higher level of job satisfaction compared with those who were only covered by new rural cooperative medical insurance. Five insurances are the most complete type of social insurance for the workers that are provided by the state and the company, while the new rural cooperative medical insurance is the most basic health social security that is purchased by rural residents themselves.

As the results of model 2 show, work experience was significantly associated with higher job satisfaction. When including work experience as an independent variable, marital status, relation with clients, and social insurance were still significantly associated with job satisfaction, while gender and ways to get jobs no longer showed significant association. Different from model 1, those who were covered by three insurances, rather than five insurances, were found to be more likely to have a higher level of job satisfaction, compared with those who were only covered by new rural cooperative medical insurance.

We also explored the correlation between job satisfaction and burnout. As Table 8 shows, emotional exhaustion and lack of personal accomplishment are negatively associated with the level of job satisfaction with very high statistical significance. Thus, it is necessary to take burnout levels into account when analyzing job satisfaction. We have carried out multivariable linear regressions to identify factors related to emotional exhaustion and lack of personal accomplishment, which showed that those who were covered by five insurances, rather than three insurances, were found to be less likely to suffer from lack of personal accomplishment, compared with those who were only covered by new rural cooperative medical insurance. The detailed analysis is included in Appendix A. The complex relations between job satisfaction, burnout, and other factors are worth more nuanced analyses in the future.

## 4. Discussion

This study scrutinizes factors that influence the job satisfaction of elderly caregivers in a home-based elderly care program, including personal characteristics, working conditions, employment status, training, relationship with clients, social protection and welfare, work experience, and burnout. These caregivers provide home-based elderly care in Shanghai. More than half work on an hourly schedule, but some still work as live-in workers. Coinciding with the findings of previous studies, most of the in-home elderly caregivers in Shanghai are rural married migrant women who have low levels of education [55]. Consistent with the results of studies on migrant workers, low levels of education play a less important role in job satisfaction [58]. Social insurance, which represents social protection and welfare from the state, plays the biggest role [59]. This result is consistent with findings of research on migrant workers that these workers from rural regions are treated as second-class citizens in cities because of the household register system in China [60]. However, marital status does affect their job satisfaction, which is different from the results of research on caregivers in other countries [38,39,40,41]. This might be a result of the influence of familialism in China [61]. Divorced rural migrant women tend to encounter stigma in their working environment, which might result in a negative perception of their job.

Moreover, our research contributes to the current discussion on caregivers’ job satisfaction by finding that social insurance plays an important role in in-home elderly caregivers’ job satisfaction, which has seldom been discussed in previous studies. Filling in this blank, the results of our model show that the job satisfaction of caregivers with Five Insurance or Three Insurance plans is higher than those with new rural cooperative medical insurance.

In China, social insurance represents the social protection and welfare provided by the state. Since 1958, the *Hukou* (household registration) system has assigned Chinese citizens either a rural or urban *Hukou* status [62]. Although migrant workers leave their village to work in cities, they are still regarded as rural residents with rural *Hukou* in this system. The socioeconomic differences between rural and urban residents have been reflected in inequalities in the welfare they receive. As an important component of welfare, rural and urban residents are often covered by quite different social insurance schemes. According to the *Hukou* system in China, rural residents only participate in new rural cooperative medical insurance, which is heavily subsidized by the government of their hometown, while urban citizens participate in Five Insurance programs (pensions, housing provident funds, work injuries, unemployment, and medical insurance), which are heavily financed by the cities in which they live [63].

Due to the household registration system, migrant workers moving from rural areas to urban regions have difficulty participating in the Five Insurance programs. Not recognized as urban citizens, they do not have urban *Hukou* and are not entitled to urban social insurance schemes [62]. Gao, Yang, and Li’s study showed that only a small proportion of rural-to-urban migrant workers with long-term contracts are allowed to participate in the Five Insurance programs [59]. These workers are often regarded as a privileged group among migrant workers. Moreover, the number of workers participating in the Three Insurance programs is also limited. These are also heavily subsided by the urban government, but only include three types of insurance: pensions and medical and unemployment insurance. As the welfare provided by the state, the Three Insurance programs is superior to the new rural cooperative medical insurance but inferior to the Five Insurance programs. Most migrant workers still participate in new rural cooperative medical insurance. Not covered by either the Five or Three Insurance programs, most migrant workers lack work and life security. Their basic work and life rights are not well-protected.

As rural-to-urban migrant workers, only a small proportion of in-home elderly caregivers participate in Five Insurance or Three Insurance programs [55]. Our research shows that most of these caregivers are still covered by new rural cooperative medical insurance. The in-home elderly caregivers participating in Five Insurance or Three Insurance programs, who have more welfare to guarantee their work security and labor rights, have a higher level of job satisfaction than those with rural cooperative medical insurance. This highlights that the relationship between job satisfaction and welfare, such as social insurance, should not be neglected by researchers.

Relationships with clients were significantly associated with in-home elderly caregivers’ job satisfaction. The results of our models show that in-home elderly caregivers who have a very good relationship with clients have a higher level of job satisfaction than those who do not have a very good relationship with clients. The results imply that a better relationship with clients might contribute to a higher level of job satisfaction among caregivers. These findings coincide with the literature on in-home caregivers’ labor process, which concludes that frequent conflict between clients and in-home caregivers might result in the distress of caregivers [60]. Home-based elderly care relies on face-to-face interaction between clients and caregivers. The clients’ strict labor control of caregivers tends to engender a feeling of alienation and burnout among caregivers [64]. Previous studies show that in-home caregivers sometimes encounter emotional abuse and sexual harassment from their clients, which leads to trauma, undermining their devotion to care work [65,66,67,68]. In contrast, the semi-familial relationship between clients and in-home caregivers might convey emotional feedback and support, which reinforces caregivers’ work identity and enthusiasm [69]. Therefore, relationships with clients play an important role in the job satisfaction of in-home elderly caregivers.

Work experience in the profession also showed significant association with in-home elderly caregivers’ job satisfaction. The model results demonstrate that those who have been working for more years in the profession are more likely to enjoy a higher level of job satisfaction [70]. This is different from the findings of research on home care in Taiwan, which revealed that caregivers with more years of working experience were more likely to have a lower level of job satisfaction. However, this coincides with the findings of other studies, which suggest that formal caregivers in hospitals with more work experience have a higher level of job satisfaction [71,72,73,74]. One reason might be that senior caregivers tend to adjust better to the work environment than their younger colleagues [71,72,73,74]. Another reason for this might be that healthcare professionals who have found more strategies to maintain their satisfaction are more likely to survive in their profession [75].

However, the results of this research show that working conditions, including income, rest days, and type of work, have no significant effect on in-home elderly caregivers’ job satisfaction in Shanghai, which is not consistent with the findings of previous studies on caregivers in institutional care systems, such as nursing homes, in other countries. In contrast to caregivers in nursing homes, most in-home elderly caregivers in Shanghai are middle-aged rural migrant women who lack opportunities for professional development and decision-making in their working environment. According to ethnographic research, these middle-aged rural migrant women are a homogeneous group whose status in the labor market is low and whose length of work is long [76,77]. Furthermore, home-based elderly care is deemed “dirty” and “bad” work, in contrast to other types of care work, such as childcare [77]. Like other migrant workers, they depend on their social network, such as relatives and friends, to seek job opportunities [78]. Their ways of finding work and their contractual status have no significant effect on their job satisfaction.

In addition, the training programs were not significantly associated with the in-home elderly caregivers’ job satisfaction. This is different from the results of previous studies, which have shown that whether companies provide caregivers with continuous training and whether the caregivers receive training are positively related to the level of job satisfaction [5,79]. In contrast, our model shows that 81.8% of respondents have received training, but the experience of receiving training is not significantly associated with the job satisfaction of the in-home elderly caregivers. Furthermore, there is also no significant association between whether the company provides training or not and the job satisfaction of in-home elderly caregivers. In addition, our research shows that the number of certificates and the in-home elderly caregivers’ job satisfaction are not significantly associated. The reason for this might be that the training they receive tends to be irrelevant to their work demands. This has been largely discussed in previous studies on in-home caregivers’ training, which reveals that the training received by in-home caregivers is often not related to their work and thus is deemed useless by caregivers [4].

Lastly, our research reveals the complex relationship between in-home elderly caregivers’ job satisfaction and their psychological status such as burnout. The results demonstrate that the level of job satisfaction is negatively associated with emotional exhaustion and lack of personal accomplishment with very high statistical significance, which implies that in-home elderly caregivers’ job satisfaction is negatively associated with their burnout level. This is consistent with the findings of previous studies, which suggest that emotional exhaustion and lack of personal accomplishment might lead to low job satisfaction [10,32], and high job satisfaction could relieve the emotional exhaustion and contribute to a low level of burnout [33,34,35].

## 5. Limitations

This study has certain limitations. First, this study used the Respondent-Driven-Sampling approach and, therefore, has the limitation inherent to this type of sampling method. Although RDS sampling is most suitable for the group of migrant workers who have no sampling frame, some sampling error is still difficult to avoid. Second, the sample is limited to Shanghai, which is one of the largest metropolises in China; therefore, the results should be complemented by research on in-home caregivers in other types of cities before being generalized to a national scale. In-home elderly caregivers in small cities are not migrant workers; therefore, their welfare, such as the social insurance scheme they participate in, is different from those in large cities such as Shanghai. However, our research could serve as a foundation for future studies on in-home elderly caregivers in other cities and even countries. Thirdly, this study has not adopted the Maslach Burnout Inventory to explore the psychometric characteristics of the respondents, although the existing items are an approximation to some of the items in MBI. Separate studies on burnout of in-home elderly caregivers can be carried out in the future using MBI.

## 6. Conclusions

Examining the job satisfaction of in-home elderly caregivers in Shanghai is essential for promoting the quality of home-based elderly care in the long-term care system and enhancing the elderly care system in developing countries. As the most important type of workers in the long-term care system, in-home elderly caregivers’ job satisfaction affects their work performance, retention, and elderly clients’ satisfaction. In our research, job satisfaction includes four aspects: satisfaction with work income, relationships with clients, employment status, and working conditions. We analyzed the relationship between job satisfaction and personal characteristics, working conditions, training, relationships with clients, welfare, work experience, and burnout. We found that, for these in-home elderly caregivers, one of the most important factors that contributes to a higher level of job satisfaction is the social protection and welfare that these migrant workers receive from the state. Coinciding with previous studies, our research shows that personal characteristics play a less-important role, while the relationship with clients and work experience have a significant influence on the job satisfaction of in-home elderly caregivers. It also found that in-home elderly caregivers’ job satisfaction is negatively associated with their burnout level.

Our results contribute to the current literature on long-term care in two dimensions. The first dimension is that improving working conditions and providing training programs may not be enough to increase the job satisfaction of in-home elderly caregivers, who are mainly middle-aged migrant women in developing countries. Another dimension is that social protection and welfare, such as social insurance, are important factors for improving the job satisfaction of in-home elderly caregivers, which may also contribute to better work performance among these workers and better quality of home-based elderly care. These two dimensions of results are relevant to policy makers such as the Ministry of Human Recourse and Social Security of the People’s Republic of China and the local government. Due to the future need of in-home elderly caregivers in the long-term care system, a more comprehensive understanding of factors affecting these caregivers’ job satisfaction would be helpful for formulating supportive policies in the elderly care sector. In addition, welfare from the state, such as social insurance, should be considered if policy makers want to enhance in-home elderly caregivers’ work performance and the quality of long-term care, particularly in large cities.

## Figures and Tables

**Table 1:** Personal characteristics and employment-related status.

Variable	
**Gender**, *n* (%)	
Women	268 (94.0)
Men	17 (6.0)
**Age**, *Median* (*SD*)	51 (5.8)
**Level of education**, *n* (%)	
No education	17 (6.0)
Primary school and under	89 (31.2)
Junior high school	136 (47.7)
High school	38 (13.3)
College and above	5 (1.8)
**Marital status**, *n* (%)	
Married	258 (90.5)
Divorced	14 (4.9)
Widowed	13 (4.6)
**Log (annual income)**, *Median* (*SD*)	10.7 (2.2)
**Number of rest days per month**, *Median* (*SD*)	1.7 (2.2)
**Type of work**, *n* (%)	
Hourly	174 (61.1)
Lived in	69 (24.2)
Day shift	42 (14.7)
**Way to obtain jobs**, *n* (%)	
Relatives and friends	140 (49.1)
Agencies	112 (39.3)
Internet	33 (11.6)
**Type of contract**, *n* (%)	
No contract	82 (28.8)
Labor contract	156 (54.7)
Tripartite agreement	42 (14.7)
Private agreement with clients	5 (1.8)
**With training experience**, *n* (%)	233 (81.8)
**Company provided training**, *n* (%)	168 (58.9)
**Number of certificates**, *Median* (*SD*)	1.6 (1.2)
**In a very good relation with clients**, *n* (%)	156 (54.7)
**Social insurance**, *n* (%)	
New rural cooperative medical insurance	250 (87.7)
Three insurances	16 (5.6)
Five insurances	19 (6.7)
**Work experience (year)**, *Median* (*SD*)	6.2 (6.5)

**Table 2:** Level of job satisfaction.

Level of Satisfaction	*n* (%)				
	1	2	3	4	5
Work income	20 (7.0)	28 (9.8)	87 (30.5)	106 (37.2)	44 (15.4)
Relationship with clients	1 (0.4)	3 (1.1)	33 (11.6)	141 (49.5)	107 (37.5)
Professional status	4 (1.4)	40 (14.0)	83 (29.1)	112 (39.3)	46 (16.1)
Working conditions	12 (4.2)	25 (8.8)	75 (26.3)	128 (44.9)	45 (15.8)

**Table 3:** Component matrix of job satisfaction (after rotation).

Variable	Factor 1	Uniqueness
Satisfaction level of work income	0.6548	0.5713
Satisfaction level of relationship with clients	0.6898	0.5242
Satisfaction level of professional status	0.7813	0.3896
Satisfaction level of working conditions	0.7689	0.4088
Eigenvalue	2.106	
Proportion	52.65%	

**Table 4:** Psychometric characteristics related to burnout.

		*n* (%)				
		Never	Seldom	Sometimes	Often	Always
**How often do you feel that… in the past month?**	I cannot have any break during the work (Lack of break)	117 (41.1)	50 (17.5)	42 (14.7)	143 (15.1)	33 (11.6)
	work has disrupted my sleep (Disruption of sleep)	171 (60.0)	31 (10.9)	36 (12.6)	20 (7.0)	27 (9.5)
	I have to adjust emotionally during the work (Need for emotional adjustment)	93 (32.6)	69 (24.2)	76 (26.7)	37 (13.0)	10 (3.5)
	work makes me feel like I’m about to break down (Breakdown)	192 (67.4)	47 (16.5)	27 (9.5)	17 (5.9)	2 (0.7)
	I have gradually lost interest in my job (Loss of interest)	202 (70.9)	37 (13.0)	25 (8.8)	16 (5.6)	5 (1.7)
		**Strongly disagree**	**Disagree**	**Neither agree or disagree**	**Agree**	**Strongly agree**
**To what extent do you agree that…**	I like my job (Liking the job)	106 (37.2)	99 (34.7)	51 (17.9)	18 (6.3)	11 (3.9)
	my job helps me meet more people (Meeting more people)	106 (37.2)	115 (40.3)	31 (10.9)	20 (7.0)	13 (4.6)

**Table 5:** Component matrix of burnout (after rotation).

Variable	Factor 1	Factor 2	Uniqueness
Lack of break	0.6422	0.0901	0.5795
Disruption of sleep	0.5739	0.1641	0.6438
Need for emotional adjustment	0.7396	0.0920	0.4446
Breakdown	0.7865	0.0609	0.3777
Loss of interest	0.6687	0.1637	0.5261
Liking the job	0.3506	0.7359	0.3355
Meeting more people	−0.0482	0.8932	0.1998
Eigenvalue	2.480	1.413	
Proportion	35.42%	20.19%	

**Table 6:** Indices of job satisfaction and burnout.

	Mean	SD
Job satisfaction	62.1	20.3
Emotional exhaustion	32.8	19.7
Lack of personal accomplishment	34.8	19.5

**Table 7:** Multiple linear regression analysis of job satisfaction.

	Model 1			Model 2		
Independent Variables	Coefficient	SE	*p*	Coefficient	SE	*p*
**Gender** (ref. women)						
men	−9.764	5.354	0.069 ^+^	−7.553	5.586	0.178
**Age**	0.390	0.243	0.109	0.364	0.260	0.162
**Education** (ref. no education)						
Primary school and under	2.758	5.202	0.597	3.048	5.190	0.558
Junior high school	3.594	5.139	0.485	4.689	5.160	0.364
High school	−0.375	5.873	0.949	1.559	5.930	0.793
College and above	−2.212	9.832	0.822	−0.866	9.847	0.930
**Marital status** (ref. married)						
Divorced	−11.78	5.298	0.027 *	−13.49	5.356	0.012 *
Widowed	−1.088	5.740	0.850	−2.703	5.762	0.639
**log (Annual income)**	0.182	0.570	0.750	0.0411	0.579	0.943
**Number of rest days per week**	0.200	0.316	0.528	0.154	0.363	0.671
**Type of work** (ref. hourly)						
Lived-in	0.650	3.487	0.852	0.909	3.501	0.795
Day shift	0.971	3.467	0.780	2.414	3.544	0.496
**Ways to get jobs** (ref. relatives and friends)						
Agencies	0.880	2.649	0.740	−0.151	2.680	0.955
Internet	6.585	3.924	0.095^+^	4.477	3.987	0.263
**Contractual status** (ref. no contract)						
Labor contract	1.402	3.482	0.688	2.710	3.584	0.450
Tripartite agreement	−3.051	3.981	0.444	−2.585	3.974	0.516
Private agreement with clients	5.361	9.760	0.583	5.817	9.743	0.551
**Training experience** (ref. none)						
Has received previous training	3.716	4.155	0.372	4.361	4.183	0.298
**Training provision** (ref. none)						
Company provided training	−1.422	2.759	0.607	−1.615	2.779	0.562
**Number of certificates**	−0.576	1.198	0.631	−1.114	1.255	0.376
**Relation with clients** (ref. not very good)						
Very good	17.60	2.443	0.000 ***	17.94	2.471	0.000 ***
**Social insurance** (ref. New rural cooperative medical insurance)						
Three insurances	8.629	5.390	0.111	9.423	5.602	0.094 ^+^
Five insurances	8.871	4.900	0.071 ^+^	6.196	5.077	0.224
**Work experience (year)**				0.449	0.214	0.037 *
**adj. R^2^**	0.175			0.187		

^+^*p* < 0.10, * *p* < 0.05, *** *p* < 0.001.

**Table 8:** Correlation and significance between job satisfaction and burnout.

	Job Satisfaction	Emotional Exhaustion	Lack of Personal Accomplishment
**Job satisfaction**	1.0000		
**Emotional exhaustion**	−0.4212 ***	1.0000	
**Lack of personal accomplishment**	−0.3641 ***	0.0000 ***	1.0000

*** *p* < 0.001.

## Data Availability

Data are not available due to confidentiality.

## Appendix A

**Table A1:** Multiple linear regression analysis of burnout.

	Model 1 Emotional Exhaustion			Model 2 Lack of Personal Accomplishment		
Independent Variables	Coefficient	SE	*p*	Coefficient	SE	*p*
**Gender** (ref. women)						
Men	−1.982	5.978	0.740	0.905	5.806	0.876
**Age**	−0.0554	0.278	0.842	−0.538	0.270	0.047 *
**Education** (ref. no education)						
Primary school and under	−5.378	5.554	0.334	0.569	5.395	0.916
Junior high school	−7.204	5.522	0.193	−1.721	5.364	0.749
High school	−2.713	6.345	0.669	1.066	6.163	0.863
College and above	−13.22	10.54	0.211	−3.665	10.24	0.721
**Marital status** (ref. married)						
Divorced	3.959	5.732	0.490	−0.621	5.567	0.911
Widowed	−0.577	6.166	0.926	1.649	5.990	0.783
**log (Annual income)**	0.0207	0.619	0.973	0.684	0.601	0.256
**Number of rest days per week**	−0.760	0.388	0.052 ^+^	0.237	0.377	0.531
**Type of work** (ref. hourly)						
Lived-in	5.143	3.747	0.171	4.116	3.639	0.259
Day shift	−0.215	3.792	0.955	−1.744	3.684	0.636
**Ways to get jobs** (ref. relatives and friends)						
Agencies	−2.411	2.868	0.401	−2.790	2.786	0.318
Internet	−2.606	4.267	0.542	4.002	4.145	0.335
**Contractual status** (ref. no contract)						
Labor contract	2.267	3.836	0.555	5.086	3.726	0.174
Tripartite agreement	3.522	4.253	0.408	2.957	4.131	0.475
Private agreement with clients	10.27	10.43	0.326	11.16	10.13	0.272
**Training experience** (ref. none)						
Has received previous training	2.098	4.476	0.640	−4.500	4.348	0.302
**Training provision** (ref. none)						
Company provided training	1.948	2.974	0.513	−6.978	2.889	0.016 *
**Number of certificates**	0.261	1.343	0.846	−1.204	1.305	0.357
**Relationship with clients** (ref. not very good)						
Very good	−4.026	2.645	0.129	−4.037	2.569	0.117
**Social insurance** (ref. New rural cooperative medical insurance)						
Three insurances	−6.585	5.995	0.273	−4.110	5.823	0.481
Five insurances	−1.663	5.433	0.760	−8.956	5.277	0.091 ^+^
**Work experience (year)**	−0.267	0.229	0.245	−0.103	0.223	0.643
**R^2^**	0.080			0.114		

^+^*p* < 0.10, * *p* < 0.05.

## References

[1] Johnson S., Bacsu J., Abeykoon H., Mclntosh T., Jeffery B., Novik N. (2018). No place like home: A systematic review of home care for older adults in Canada. Can. J. Aging/La Rev. Can. Du Vieil..

[2] Genet N., Boerma W., Kringos D., Bouman A., Francke A., Fagerström C., Melchiorre M., Greco C., Devillé W. (2011). Home care in Europe: A systematic literature review. BMC Health Serv. Res..

[3] Pangaribowo E.H., Keban Y.T., Darwin M. (2020). Elderly care: A study on community care services in Sleman, DIY, Indonesia. J. Aging Res..

[4] Iecovich E. (2011). What makes migrant live-in home care workers in elder care be satisfied with their job?. Gerontologist.

[5] Pardo-Garcia I., Martinez-Lacoba R., Escribano-Sotos F. (2021). Socioeconomic Factors Related to Job Satisfaction among Formal Care Workers in Nursing Homes for Older Dependent Adults. Int. J. Environ. Res. Public Health.

[6] Rodrigo-Baños V., Moral-Pairada M.d., González-de L. (2021). A Comprehensive Assessment of Informal Caregivers of Patients in a Primary Healthcare Home-Care Program. Int. J. Environ. Res. Public Health.

[7] Peña-Longobardo L.M., Peña-Longobardo M., Oliva-Moreno J., Larrañaga-Padilla I., García-Calvente M.M. (2021). Health, Work, and Social Problems in Spanish Informal Caregivers: Does Gender Matter? (The CUIDAR-SE Study). Int. J. Environ. Res. Public Health.

[8] Rajamohan S., Porock D., Chang Y. (2019). Understanding the relationship between staff and job satisfaction, stress, turnover, and staff outcomes in the personal centered care nursing home arena. J. Nurs. Scholarsh..

[9] Dilig-Ruiz A., MacDonald I., Varin M.D., Vandyk A., Graham I.D., Squires J.E. (2018). Job satisfaction among critical care nurses: A systematic review. Int. J. Nurs. Stud..

[10] Chen X., Ran L., Zhang Y., Yang J., Yao H., Zhu S., Tan X. (2019). Moderating role of job satisfaction on turnover intention and burnout among workers in primary care institutions: Across-sectionalstudy. BMC Public Health.

[11] United Nations (2019). World Population Ageing 2019.

[12] United Nations (2015). World Population Ageing 2015.

[13] Yang W., Wu B., Tan S., Li B., Lou V.Q., Zhou C., Chen X., Fletcher J.R., Carrino L., Hu B. (2021). Understanding health and social challenges for aging and long-term care in China. Res. Aging.

[14] Chen F., Liu G. (2009). Population aging in China. International Handbook of Population Aging 2009.

[15] Shang X., Wu X. (2011). The care regime in China: Elder and child care. J. Comp. Soc. Welf..

[16] Zeng L., Zhu X., Meng X., Mao Y., Wu Q., Shi Y., Zhou L. (2014). Responsibility and burden from the perspective of seniors’ family caregivers: A qualitative study in Shanghai, China. Int. J. Clin. Exp. Med..

[17] Wu B., Carter M., Goins R., Cheng C. (2005). Emerging services for community-based long-term care in urban China: A systematic analysis of Shanghai’s community-based agencies. J. Aging Soc. Policy.

[18] Wong Y.C., Leung J. (2012). Long-term care in China: Issues and prospects. J. Gerontol. Soc. Work.

[19] Fang E.F., Xie C., Schenkel J.A., Wu C., Long Q., Cui H., Aman Y., Frank J., Liao J., Zou H. (2020). A research agenda for ageing in China in the 21st century: Focusing on basic and translational research, long-term care, policy and social networks. Ageing Res. Rev..

[20] Fu Y.Y., Chui E.W.T. (2020). Determinants of patterns of need for home and community-based care services among community-dwelling older people in urban China: The role of living arrangement and Filial Piety. J. Appl. Gerontol..

[21] Zhu Y., Österle A. (2017). Rural-urban disparities in unmet long-term care needs in China: The role of the hukou status. Soc. Sci. Med..

[22] Meng D., Xu G., Davidson M. (2021). Perceived unmet needs for community-based long-term care services among urban older adults: A cross sectional study. Geriatr. Nurs..

[23] Gu D., Vlosky D. (2008). Long-term care needs and related issues in China. Soc. Sci. Health Care Med..

[24] Hu B. (2019). Projecting future demand for informal care among older people in China: The road towards a sustainable long-term care system. Health Econ. Policy Law.

[25] Zeng Y., Hu X., Li Y., Zhen X., Gu Y., Sun X., Dong H. (2019). The quality of caregivers for the elderly in long-term care institutions in Zhejiang Province, China. Int. J. Environ. Res. Public Health.

[26] Engström M., Ljunggren B., Lindqvist R., Carlsson M. (2006). Staff satisfaction with work, perceived quality of care and stress in elderly care: Psychometric assessments and associations. J. Nurs. Manag..

[27] Hodson R. (2005). Management behaviour as social capital: A systematic analysis of organizational ethnographies. Br. J. Ind. Relat..

[28] Judge T.A., Thoresen C.J., Bono J.E., Patton G.K. (2001). The job satisfaction-job performance relationship: A qualitative and quantitative review. Psychol. Bull..

[29] Chao C., Ku P., Wang Y., Lin Y. (2016). The effects of job satisfaction and ethical climate on service quality in elderly care: The case of Taiwan. Total Qual. Manag. Bus. Excell..

[30] Brewer C.S., Kovner C., Greene W., Tukov-Shuser M., Djukic M. (2012). Predictors of actual turnover in a national sample of newly licensed registered nurses employed in hospitals. J. Adv. Nurs..

[31] Maslach C., Jackson S.E. (1981). The measurement of experienced burnout. J. Organ. Behav..

[32] Kalliath T., Morris R. (2002). Job satisfaction among nurses: A predictor of burnout levels. JONA J. Nurs..

[33] Queiros C., Carlotto M.S., Kaiseler M., Dias S., Pereira A.M. (2013). Predictors of burnout among nurses: An interactionistapproach. Psicothema.

[34] Liang Y., Zhou S.P., Gao Y.S., Wang X.D. (2020). Analysis on the Influencing Factors of Job Burnout of Nurses in Haikou Tertiary Hospital. Open J. Nurs..

[35] Wang H., Jin Y., Wang D., Zhao S., Sang X., Yuan B. (2020). Job satisfaction, burnout, and turnover intention among primary care providers in rural China: Results from structural equation modeling. BMC Fam. Pract..

[36] Best M.F., Thurston N.E. (2004). Measuring nurse job satisfaction. JONA J. Nurs. Adm..

[37] Coomber B., Barriball K.L. (2007). Impact of job satisfaction components on intent to leave and turnover for hospital-based nurses: A review of the research literature. Int. J. Nurs. Stud..

[38] Denton M., Zeytinoglu I., Kusch K., Davies S. (2007). Market-modelled home care: Impact on job satisfaction and propensity to leave. Can. Public Policy.

[39] Ejaz F.K., Noelker L.S., Menne H.L., Bagaka J. (2008). The impact of stress and support on direct care workers’ job satisfaction. Gerontologist.

[40] Friedman S.M., Daub C., Gresci K., Keyser R. (1999). A comparison of job satisfaction among nursing assistants in nursing homes and the Program of All-Inclusive Care for the Elderly (PACE). Gerontologist.

[41] Royse D., Dhooper S.S., Howard K. (1988). Job satisfaction among home health aides. Home Health Care Serv. Q..

[42] Laschinger H.K.S., Zhu J., Read E. (2016). New nurses’ perceptions of professional practice behaviours, quality of care, job satisfaction and career retention. J. Nurs. Manag..

[43] Begat I., Ellefsen B., Severinsson E. (2005). Nurses’ satisfaction with their work environment and the outcomes of clinical nursing supervision on nurses’ experiences of well-being—A Norwegian study. J. Nurs. Manag..

[44] Delp L., Wallace S., Geiger-Brown J., Carles M. (2010). Job stress and job satisfaction: Home Care Workers in a consumer-Directed model of care. Health Serv. Res..

[45] Denton M., Zeytinoglu I.U., Davies S., Lian J. (2002). Job stress and job dissatisfaction of home care workers in the context of health care restructuring. Int. J. Health Serv..

[46] Buelow J.R., Winburn K., Hutcherson J. (1999). Job satisfaction of home care assistants related to managerial practices. Home Health Care Serv. Q..

[47] Morris L. (2009). Quits and job changes among home care workers in Maine: The role of wages, hours, and benefits. Gerontologist.

[48] Laschinger H.K.S. (2008). Effect of empowerment on professional practice environments, work satisfaction, and patient care quality: Further testing the nursing worklife model. J. Nurs. Care Qual..

[49] Coogle C.L., Parham I.A., Jablonski R., Rachel J.A. (2007). Enhanced care assistant training to address the workforce crisis in home care: Changes related to job satisfaction and career commitment. Care Manag. J..

[50] Xu Q., Chow J.C. (2011). Exploring the community-based service delivery model: Elderly care in China. Int. Soc. Work.

[51] Sheng X., Settles B.H. (2006). Intergenerational relationships and elderly care in China: A global perspective. Curr. Sociol..

[52] Eustis N.N., Fischer L.R. (1991). Relationships between home care clients and their workers: Implications for quality of care. Gerontologist.

[53] Feldman H., Sapienza A.M., Kane N.M. (1990). Who Cares for Them?: Workers in the Home Care Industry.

[54] Benjamin A., Matthias R., Franke T.M. (2000). Comparing consumer-directed and agency models for providing supportive services at home. Health Serv. Res..

[55] Jilili M., Liu L., Yang A. (2021). The impact of perceived discrimination, positive aspects of caregiving on depression among caregivers: Mediating effect of job satisfaction. Curr. Psychol..

[56] Xing Z. (2021). The “hyper precarity” of informal employment under the COVID-19 pandemic and workers’ coping strategies: Taking domestic workers in Beijing as an Example. J. Chin. Women’s Stud..

[57] Burgoon B., Rooduijn M. (2021). Immigrationalization of welfare politics? Anti-immigration and welfare attitudes in context. West Eur. Politics.

[58] To S.M., Tam H.L. (2014). Generational differences in work values, perceived job rewards, and job satisfaction of Chinese female migrant workers: Implications for social policy and social services. Soc. Indic. Res..

[59] Gao Q., Yang S., Li S. (2012). Labor contracts and social insurance participation among migrant workers in China. China Econ. Rev..

[60] Fu H., Su Y., Ni A. (2018). Selling motherhood: Gendered emotional labor, citizenly discounting, and alienation among China’s migrant domestic workers. Gend. Soc..

[61] Yan Y. (2018). Neo-familism and the state in contemporary China. Urban Anthropol. Stud. Cult. Syst. World Econ. Dev..

[62] Cheng Z., Guo F., Hugo G., Yuan X. (2013). Employment and wage discrimination in the Chinese cities: A comparative study of migrants and locals. Habitat Int..

[63] You X., Kobayashi Y. (2009). The new cooperative medical scheme in China. Health Policy.

[64] Parreñas R.S., Kantachote K., Silvey R. (2021). Soft violence: Migrant domestic worker precarity and the management of unfree labour in Singapore. J. Ethn. Migr. Stud..

[65] Anderson B. (1997). Servants and slaves: Europe’s domestic workers. Race Cl..

[66] Huang S., Yeoh B.S.A. (2007). Emotional labour and transnational domestic work: The moving geographies of ‘maid abuse’ in Singapore. Mobilities.

[67] Pan S.-M., Yang J. (2012). Outsiders in the family: Abuse of migrant domestic Workers in Taiwan. Asian J. Women’s Stud..

[68] Corossacz V.R. (2019). Sexual harassment and assault in domestic work: An exploration of domestic workers and union organizers in Brazil. J. Lat. Am. Caribb. Anthropol..

[69] Lan P. (2006). Global Cinderellas: Migrant Domestics and Newly Rich Employers in Taiwan.

[70] Chou Y.-C., Fu L., Kröger T., Ru-Yan C. (2011). Job satisfaction and quality of life among home care workers: A comparison of home care workers who are and who are not informal carers. Int. Psychogeriatr..

[71] Khamlub S., Harun-Or-Rashid M.D., Sarker M.A.B., Hirosawa T., Outavong P., Sakamoto J. (2013). Job satisfaction of health-care workers at health centers in Vientiane Capital and Bolikhamsai Province, Lao PDR. Nagoya J. Med. Sci..

[72] Al Otabi M., Shah M.A., Chowdhury R.I., Al-Enezi N. (2004). Determinants of job satisfaction among nurses in Kuwait. Aust. J. Adv. Nurs..

[73] Fung-Kam L. (1998). Job satisfaction and autonomy of Hong Kong registered nurses. J. Adv. Nurs..

[74] Chien W.T., Yick S.Y. (2016). An investigation of nurses’ job satisfaction in a private hospital and its correlates. Open Nurs. J..

[75] Ommen O., Driller E., Köhler T., Kowalski C., Ernstmann N., Neumann M., Steffen P., Pfaff H. (2009). The relationship between social capital in hospitals and physician job satisfaction. BMC Health Serv. Res..

[76] Gaetano A.M., Jacka T. (2004). On the Move: Women and Rural-to-Urban Migration in Contemporary China.

[77] Tong X. (2018). Gendered labour regimes: On the organizing of domestic workers in urban China. Asian J. Ger. Eur. Stud..

[78] Zhang N. (2011). The impact of guanxi networks on the employment relations of rural migrant women in contemporary China. Ind. Relat. J..

[79] Vernooij-Dasssen M.J., Faber M., Rikkert M., Koopmans R., Achterberg T., Braat D., Rass G., Wollersheim H. (2009). Dementia care and labour market: The role of job satisfaction. Aging Ment. Health.

